# The lack of correlation between proliferation (Ki-67, PCNA, LI, Tpot), p53 expression and radiosensitivity for head and neck cancers

**DOI:** 10.1038/sj.bjc.6690535

**Published:** 1999-07

**Authors:** T Björk-Eriksson, C M L West, E Cvetskovska, M Svensson, E Karlsson, B Magnusson, N J Slevin, S Edström, C Mercke

**Affiliations:** Departments of Oncology, Otorhinolaryngology Head and Neck Surgery and Pathology, Sahlgrenska University Hospital, Gothenburg, S-413 45, Sweden; Department of Oral Pathology, Faculty of Odontology, University of Gothenburg, Sweden; Christie (CRC) Research Centre, Wilmslow Road, Manchester, M20 4BX, UK

**Keywords:** proliferation, p53, radiosensitivity, head and neck cancer

## Abstract

A study was made of the relationship between measurements of radiosensitivity versus proliferation and p53 status in head and neck cancers. Inherent tumour radiosensitivity was assessed as surviving fraction at 2 Gy (SF2) using a clonogenic soft agar assay (*n* = 77). The results were compared to data on proliferation obtained by both flow cytometry (labelling index (LI), the potential doubling time (Tpot) *n* = 55) and immunohistochemistry (Ki-67 and PCNA; *n* = 68), together with immunohistochemical p53 expression (*n* = 68). There were no overall significant differences in the median values of the various parameters analysed for the different sites within the head and neck region, disease stages, grades of tumour differentiation or nodal states. A subgroup analysis showed that oropharyngeal (*n* = 22) versus oral cavity (*n* = 35) tumours were more radiosensitive (*P* = 0.056) and had a higher Ki-67 index (*P* = 0.001). Node-positive tumours had higher LI (*P* = 0.021) and a trend towards lower Tpot (*P* = 0.067) values than node-negative ones. No correlations were seen between SF2 and any of the parameters studied. The long-standing dogma of an increased radiosensitivity of rapidly proliferating cells in contrast to slowly proliferating cells was not confirmed. The study shows that parallel measurements of different biological markers can be obtained for a large number of patients with head and neck cancers. The independence of the various parameters studied suggests that there may be potential for their combined use as prognostic factors for the outcome of radiotherapy. © 1999 Cancer Research Campaign


*British Journal of Cancer* (1999) **80**(9), 1400–1404
© 1999 Cancer Research Campaign
Article no. bjoc.1999.0535

### The lack of correlation between proliferation (Ki-67, PCNA, LI, Tpot), p53 expression and radiosensitivity for head and neck cancers

**T Björk-Eriksson[1], CML West[5], E Cvetskovska[2], M Svensson[3], E Karlsson[1], B Magnusson[4], NJ Slevin[5], S Edström[2] and C Mercke[1]**

Departments of [1]Oncology, [2]Otorhinolaryngology Head and Neck Surgery and [3]Pathology, Sahlgrenska University Hospital, S-413 45, Gothenburg, Sweden; [4]Department of Oral Pathology, Faculty of Odontology, University of Gothenburg, Sweden; [5]Christie (CRC) Research Centre, Wilmslow Road, Manchester M20 4BX, UK

**Summary** A study was made of the relationship between measurements of radiosensitivity versus proliferation and p53 status in head and neck cancers. Inherent tumour radiosensitivity was assessed as surviving fraction at 2 Gy (SF2) using a clonogenic soft agar assay ($n$ = 77). The results were compared to data on proliferation obtained by both flow cytometry (labelling index (LI), the potential doubling time (Tpot) $n$ = 55) and immunohistochemistry (Ki-67 and PCNA; $n$ = 68), together with immunohistochemical p53 expression ($n$ = 68). There were no overall significant differences in the median values of the various parameters analysed for the different sites within the head and neck region, disease stages, grades of tumour differentiation or nodal states. A subgroup analysis showed that oropharyngeal ($n$ = 22) versus oral cavity ($n$ = 35) tumours were more radiosensitive ($P$ = 0.056) and had a higher Ki-67 index ($P$ = 0.001). Node-positive tumours had higher LI ($P$ = 0.021) and a trend towards lower Tpot ($P$ = 0.067) values than node-negative ones. No correlations were seen between SF2 and any of the parameters studied. The long-standing dogma of an increased radiosensitivity of rapidly proliferating cells in contrast to slowly proliferating cells was not confirmed. The study shows that parallel measurements of different biological markers can be obtained for a large number of patients with head and neck cancers. The independence of the various parameters studied suggests that there may be potential for their combined use as prognostic factors for the outcome of radiotherapy.

**Keywords:** proliferation; p53; radiosensitivity; head and neck cancer

Head and neck cancer is a disease which is usually treated with radiotherapy, surgery alone or a combination of surgery and pre- or post-operative radiotherapy. Treatment decisions are usually based not only on results from previous trials, patient parameters (performance status, age, weight loss, other co-existing diseases) and clinicopathological factors (site, TNM status, grade of differentiation) but also on local treatment traditions. The latter will favour either radical radiotherapy, surgery, or a combined modality approach. The advantages of radiotherapy over surgery in the management of the disease is in the possibility of organ preservation and maintenance of important functions (e.g. speaking, swallowing). The advantages of surgery are short overall treatment times and less radiotherapy-specific morbidity (e.g. dry mouth, taste alteration and radionecrosis). The potential value of finding biological predictive factors is in being able to offer an individualized approach to treatment to select the most appropriate type of therapy. In theory this should lead to not only increases in local control but also reductions in treatment-specific morbidity.

Regarding biological predictive factors for radiotherapy response, tumour proliferation is probably the most widely studied parameter, in particular the potential doubling time (Tpot) of a tumour (Bourhis et al, 1993; Begg et al, 1995; Zackrisson et al, 1997). However, a recent meta-analysis of 210 aneuploid tumours treated with conventional radiotherapy showed that labelling index (LI) but not Tpot was a significant predictor of local control (Begg, 1997). Nevertheless, there are statistical and methodological questions regarding the appropriateness of pooling multicentre data and using Tpot as a continuous variable (Coucke et al, 1998). There is, therefore, still interest in measuring proliferation in head and neck cancers.

Another potential biological prognostic parameter for radiotherapy response is tumour radiosensitivity, usually measured as surviving fraction at 2 Gy (SF2) (West, 1995). There is good evidence that in-vitro clonogenic assays reflect the radioresponsiveness of tumour in vivo (reviewed in West, 1995). In particular, in cervix tumours SF2 has been shown to be an independent prognostic factor for radiotherapy response (West et al, 1997). The results from studies on head and neck cancer, however, have been disappointing. SF2 measured using the CAM (cell adhesive matrix) assay on tumour specimens from patients treated by radiotherapy and surgery showed a trend for patients with in vitro radioresistant versus -sensitive tumours to have a worse outcome but the difference was not significant (Brock et al, 1990). Using the same assay, a study on patients treated predominantly by radiotherapy alone showed that tumour radiosensitivity was significantly associated with treatment outcome (Girinsky et al, 1994) but in a recent update of the data the significance was lost (Eschwege et al, 1997). Thus, it is yet to be established whether

*Received 17 September 1998*
*Revised 12 January 1999*
*Accepted 27 January 1999*

*Correspondence to:* T Björk-Eriksson radiosensitivity measurements obtained using a clonogenic assay are prognostic for radiotherapy outcome in head and neck cancers. Although we have carried out a large study measuring tumour radiosensitivity using a soft agar assay (Björk-Eriksson et al, 1998), analysis with respect to treatment outcome awaits adequate patient follow-up.

The *TP53* gene is important in the pathogenesis of cancer and mutations in it are the most common found in human cancers. The *TP53* gene also has a clear role to play in regulating cell proliferation and radiosensitivity and as a checkpoint protein transactivating other genes (Yin et al, 1992; Bristow et al, 1996). With the possible exceptions of laryngeal- and oropharyngeal carcinomas (Sauter et al, 1992; Bradford et al, 1995; Narayana et al, 1998), most studies using immunohistochemistry have not shown any correlation between p53 overexpression and local control or survival in head and neck cancers (Field et al, 1993; Raybaud-Diogene et al, 1997). Therefore, it is of interest to study the relationship between p53 overexpression versus inherent tumour radiosensitivity and proliferation.

There is little information on the relationship between radiosensitivity and proliferation in the same patient, and between SF2 and p53 expression in primary tumours. Therefore, the aims of this study were to examine the relationship between SF2, various proliferation parameters (Ki-67, PCNA, LI, Tpot) and p53 expression for a large number of patients with head and neck cancers.

### MATERIALS AND METHODS

### Specimens

All tumour material was obtained following informed consent. Biopsy or surgical specimens were taken from patients with previously histopathologically confirmed and untreated primary squamous cell- or undifferentiated carcinoma of the head and neck region. Samples were collected for radiosensitivity testing, flow-cytometric analysis and routine histopathology, including later immunohistochemical staining. The specimens were divided and portions were placed in transport medium (Björk-Eriksson et al, 1998), 70% ethanol and 4% formaldehyde for the various analyses to be carried out later. All the material was from primary lesions and no selection criteria were used regarding tumour site of origin within the head and neck region or TNM-stage (Spiessl et al, 1990). The specimens were collected from the Department of Otorhinolaryngology Head and Neck Surgery, Sahlgrenska University Hospital in Gothenburg, Sweden.

### Radiosensitivity

Tumour radiosensitivity was measured on primary biopsies as the surviving fraction following an acute exposure of 2 Gy in vitro (SF2) using a soft agar clonogenic assay. The materials and methods used have been described in detail elsewhere together with a description of the immunohistochemical characterization of the malignant epithelial origin of colonies (Björk-Eriksson et al, 1998).

### Immunohistochemistry

Formalin-fixed and paraffin-embedded sections, 5-μm thick, were stained using the DAKO Techmate500–1000™ automated system. The following antigens (all DAKO™) and dilutions were used:

**Table 1** Description of clinical parameters

| Parameter | Group | *n* |
|---|---|---|
| Sex | Male | 59 |
| | Female | 18 |
| Site | Oral | 35 |
| | Oropharynx | 22 |
| | Nasopharynx | 8 |
| | Hypopharynx | 3 |
| | Larynx | 7 |
| | Sinonasal | 2 |
| Stage | II | 8 |
| | III | 14 |
| | IV | 54 |
| Nodes | Negative (N[a] = 0) | 44 |
| | Positive (N = 1–3) | 32 |
| Histology | SCC | 77 |
| | Poor | 22 |
| | Moderate | 38 |
| | Well | 7 |
| | Undifferentiated | 3 |

[a]Refers to TNM classification according to UICC. SCC, squamous cell carcinoma.

**Table 2** Summary of data

| Variable | *n* | Mean ± 1 s.d. | Median | Range |
|---|---|---|---|---|
| Age (years) | 77 | 63 ± 14 | 63 | 19–94 |
| SF2 | 77 | 0.46 ± 0.21 | 0.40 | 0.10–1.00 |
| p53 (%) | 68 | 18 ± 27 | 2 | 0–85 |
| Ki-67 (%) | 67 | 21 ± 14 | 18 | 2–60 |
| PCNA (%) | 68 | 21 ± 23 | 14 | 0–92 |
| LI (%) | 55 | 8 ± 5 | 7 | 1–24 |
| Tpot (h) | 50 | 128 ± 117 | 107 | 11–660 |

1/500 anti-human p53 mouse monoclonal antibody (clone DO-7), 1/100 anti-Ki-67 rabbit polycolonal antibody (reactivity similar to MIB-1), and 1/50 anti-PCNA mouse monoclonal antibody (clone PC-10). A microwave antigen retrieval step was used for p53 and Ki-67 only. Both primary and secondary antibodies were incubated for 25 min at room temperature. For Ki-67 and PCNA, normal human pharyngeal tonsil was used as a positive control, and for p53, a known positive human rectal carcinoma served as the positive control. The presence of carcinoma and the number of immunohistochemically stained cells were scored under a light microscope by two independent observers using a 100 squared grid and a magnification of ×400. All viable nucleated tumour cells at the 121 crossings of the grid were counted and scored as positive or negative. When there was sufficient tumour material, this procedure was repeated five times for each observer to give a total of up to 1210 cells scored. The percentage of positive nuclei was used as a measure of antigen expression.

### Flow cytometry

Administration of bromodeoxyuridine (BrdU) was carried out following approval from the Ethical Committee of the University of Gothenburg, Sweden. A short time intravenous (i.v.) infusion of 250 mg BrdU dissolved in 100 ml natural saline was given approximately 6 h before biopsy. Nuclei extraction, DNA staining, analysis on the FACS scan flow cytometer (Becton-Dickson) and

© 1999 Cancer Research Campaign

**Table 3** Distribution within the different tumour sites of the head and neck

| Site | SF2 | p53 (%) | Ki-67 (%) | PCNA (%) | LI (%) | Tpot (h) |
|---|---|---|---|---|---|---|
| Oral cavity | 0.51 ± 0.23 | 19 ± 27 | 15 ± 12 | 21 ± 24 | 7 ± 5 | 121 ± 82 |
| *n* = | 35 | 31 | 31 | 31 | 24 | 20 |
| Oropharynx | 0.38 ± 0.19 | 16 ± 27 | 27 ± 14 | 25 ± 21 | 9 ± 5 | 124 ± 116 |
| *n* = | 22 | 21 | 21 | 21 | 14 | 13 |
| Nasopharynx | 0.45 ± 0.18 | 17 ± 25 | 21 ± 14 | 5 ± 7 | 7 ± 4 | 173 ± 218 |
| *n* = | 8 | 5 | 5 | 5 | 7 | 7 |
| Hypopharynx | 0.28 ± 0.06 | 18 ± 30 | 35 ± 10 | 37 ± 41 | 16 ± 7 | 32 ± 24 |
| *n* = | 3 | 3 | 3 | 3 | 3 | 3 |
| Larynx | 0.45 ± 0.12 | 8 ± 13 | 23 ± 11 | 16 ± 12 | 8 ± 5 | 154 ± 105 |
| *n* = | 7 | 6 | 5 | 6 | 5 | 5 |
| Sinonasal | 0.68 ± 0.37 | 77 ± 1 | 26 ± 34 | 4 ± 3 | 6 ± 2 | 144 ± 60 |
| *n* = | 2 | 2 | 2 | 2 | 2 | 2 |

All values represent mean ± one standard deviation.

**Table 4** Subset analysis according to stage (II, III and IV) and tumour differentiation (Diff) (Poor, Mod and Well)

| Stage/ Diff. | SF2 | p53 (%) | Ki-67 (%) | PCNA (%) | LI (%) | Tpot (h) |
|---|---|---|---|---|---|---|
| II | 0.59 ± 0.23 | 12 ± 18 | 13 ± 8 | 12 ± 13 | 5 ± 4 | 120 ± 13 |
| *n* = | 8 | 7 | 7 | 7 | 3 | 2 |
| III | 0.40 ± 0.15 | 27 ± 33 | 23 ± 16 | 13 ± 19 | 8 ± 4 | 109 ± 76 |
| *n* = | 14 | 13 | 13 | 13 | 10 | 7 |
| IV | 0.44 ± 0.20 | 17 ± 27 | 22 ± 14 | 24 ± 24 | 8 ± 5 | 133 ± 127 |
| *n* = | 54 | 47 | 46 | 47 | 41 | 40 |
| Poor | 0.46 ± 0.20 | 15 ± 26 | 25 ± 17 | 21 ± 20 | 95 | 121 ± 114 |
| *n* = | 22 | 20 | 20 | 20 | 16 | 15 |
| Mod | 0.44 ± 0.22 | 26 ± 31 | 21 ± 14 | 21 ± 21 | 8 ± 5 | 119 ± 83 |
| *n* = | 38 | 34 | 34 | 34 | 25 | 21 |
| Well | 0.44 ± 0.18 | 5 ± 8 | 17 ± 11 | 33 ± 38 | 12 ± 9 | 73 ± 52 |
| *n* = | 7 | 7 | 7 | 7 | 4 | 4 |

All values represent mean ± one standard deviation. Stage II, III and IV refers to the TNM classification according to UICC (Spiessl et al, 1990). Tumour differentiation: Poor = poorly differentiated squamous cell carcinoma; Mod = moderately differentiated squamous cell carcinoma; Well = well-differentiated squamous cell carcinoma.

the calculations of LI as well as Tpot were performed as described previously (Lydén et al, 1995).

### Statistical analysis

Correlations between variables were determined using Spearman's rank correlation. Subset analyses were carried out with groups of more than 30 patients, which had 80% power to detect a significant correlation. The Mann–Whitney *U*-test and Kruskal–Wallis one-way analysis of variance were used to test for the level of significance between independent variables. A significance level of 0.05 was used throughout.

### RESULTS

SF2 values were obtained for 77 patients with head and neck malignancies. Table 1 summarizes the distribution of the patients according to sex, site, stage, nodal status and histology. Staging information was not available for one patient and seven tumours were classified as squamous cell carcinoma only with no further

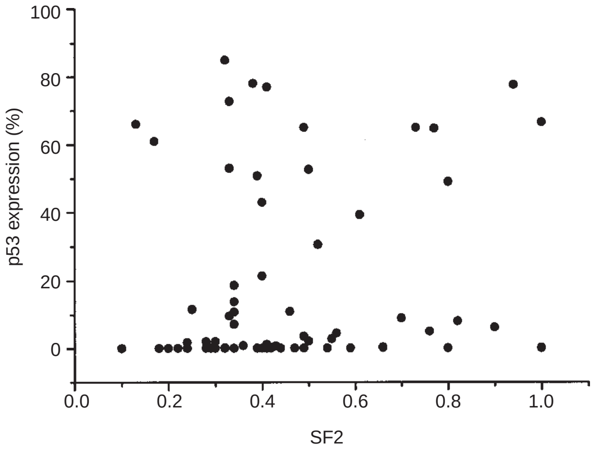

**Figure 1** The lack of relationship between tumour radiosensitivity (SF2) and the percentage of cells stained p53 positive for 68 head and neck tumours ($r = 0.17$, $P = 0.17$)

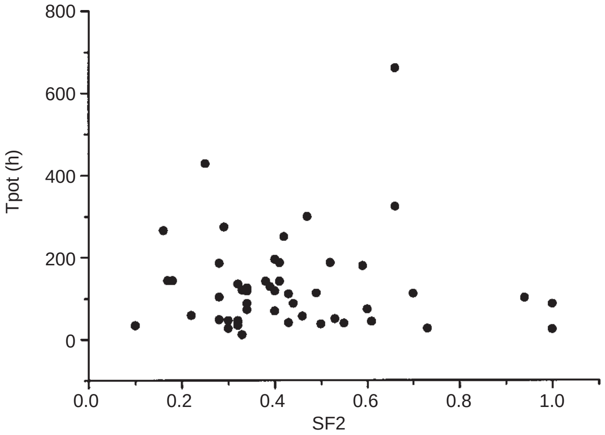

**Figure 2** The lack of relationship between tumour radiosensitivity (SF2) and proliferation (Tpot) for 50 cancers of the head and neck ($r = 0.07$, $P = 0.64$)

indication of the degree of differentiation. Table 2 summarizes the data for tumour radiosensitivity (SF2), patient age and the various immunohistochemical (p53, Ki-67, PCNA) and flow cytometry (LI, Tpot) parameters. Immunohistochemical scoring of the three antibodies studied were well-correlated for the two independent observers: p53 ($r = 0.90$, $P < 0.001$), Ki-67 ($r = 0.81$, $P < 0.001$) and PCNA ($r = 0.88$, $P < 0.001$). In contrast, there was considerable inter-tumour variability in immunohistochemical expression with coefficients of variation of 150%, 67% and 151% for p53, Ki-67 and PCNA respectively.

Before correlations between variables were obtained, an analysis was made of the distribution of values within the different sites of the head and neck region, disease stages, grades of tumour differentiation and tumour nodal status. Using the Kruskal–Wallis non-parametric test, there were no significant differences in the median values of the various parameters for the different sites within the head and neck region (Table 3). A subgroup analysis was made of the two most frequently-presenting sites. Oropharyngeal tumours were more radiosensitive ($P = 0.056$) and had a higher Ki-67 index ($P < 0.001$) than carcinomas of the oral cavity. There were no significant differences in the median values of the various parameters for the different disease stages (Table 4; $P > 0.14$) and grades of

 © 1999 Cancer Research Campaign tumour differentiation (Table 5; $P > 0.44$). Node-positive tumours had higher values for LI ($P = 0.021$) and tended to have lower values for Tpot ($P = 0.067$) than node-negative cancers.

The relationships between variables were investigated using Spearman's rank correlation. No significant correlations were seen between SF2 and Ki-67 index ($r = -0.19$, $P = 0.13$), PCNA index ($r = -0.10$, $P = 0.40$), or LI ($r = -0.10$, $P = 0.46$). The Figures illustrate the lack of relationship between SF2 versus p53 expression (Figure 1) and Tpot (Figure 2). The analyses were repeated for the largest clinical subgroups. No significant correlations were also seen when the data were reanalysed for oral cavity ($P > 0.061$), stage IV disease ($P > 0.49$) or moderately differentiated tumours ($P > 0.28$) only.

Significant correlations were seen between some of the other parameters. As expected, LI and Tpot were highly significantly correlated ($n = 50$, $P < 0.001$). A significant correlation was also seen between the expression of the two proliferative markers, Ki-67 and PCNA ($n = 67$, $P = 0.036$). There were no significant relationships between the level of p53 expression and any of the proliferation parameters.

### DISCUSSION

The individualization of radiotherapy using biological predictive tests alongside the traditional patient and clinicopathological parameters could lead to not only increased local control, but also less morbidity. The prognostic factors of most interest are those which are known to affect radiotherapy outcome, namely tumour proliferation, radiosensitivity and oxygenation, and the intrinsic radiosensitivity of normal cells. There have now been numerous studies that have examined the potential of all these parameters as prognostic factors for radiotherapy outcome (Begg, 1997). It may be, however, that the greater discrimination of radiotherapy outcome groups will be via multiple biological tests. For example, large differences in survival and local control probabilities have been shown by combining two biological parameters (Levine et al, 1995; Raybaud-Diogene et al, 1997; West et al, 1998). It is of interest, therefore, to examine the relationship between potential prognostic factors in order to show both their independence and the feasibility of carrying out multiple measurements. It is surprising then that there are little data where measurements have been made of SF2 and Tpot on primary tumours form the same individual. A paper, where Tpot and SF2 had been obtained on only nine patients, highlighted some of the problems in carrying out parallel measurements such as inadequate sample size and patient refusal (Eschwege et al, 1997).

In the work described here, tumour Tpot measurements were made on 50 out of 77 (65%) patients for whom SF2 data were collected. Reasons for failure to obtain Tpot data were predominately related to patient refusal and age. Nevertheless, we have clearly demonstrated that it is feasible to make multiple biological measurements. In addition, our work has shown that there is no relationship between the intrinsic radiosensitivity of a tumour and its rate of proliferation, whether measured by flow cytometry or the simpler immunohistochemical methods. This finding is supported by work carried out on cells in culture which has shown that there is no relationship between intrinsic radiosensitivity and the in vitro proliferation rate measured either as cell doubling time (Pekkola-Heino et al, 1994) or as LI and Tpot (Warenius et al, 1994). The lack of relationship between SF2 and Tpot/LI is encouraging for the future parallel assessment of radiosensitivity and proliferation. It has been suggested that it may be necessary to correct for differences in tumour radiosensitivity before tests based on other radiobiological parameters may have clinical significance (Tucker and Thames, 1989). It will, therefore, be of interest in the future to examine the prognostic significance of LI (and the other proliferation measures) after allowing for differences in tumour radiosensitivity.

Over the past decade there has been considerable interest in measuring the tumour suppressor and regulatory gene p53 product. In cancers of the head and neck, overexpression of p53 has been studied in relation to local control (Wilson et al, 1995; Narayana et al, 1998), survival (Sauter et al, 1992; Field et al, 1993; Nylander et al, 1995) and lately organ preservation (Bradford et al, 1995). In general, p53 expression using immunohistochemistry is not a significant prognostic factor for radiotherapy outcome and the use of functional assay has been suggested (Bristow et al, 1996). However, there is still interest in studying the relationship between tumour p53 expression and radiosensitivity as the findings have been equivocal. In a series of 24 head and neck cancer cell lines, Brachman et al (1993) found no relationship between p53 mutations and SF2. However, in 16 oral cavity carcinoma cell lines, the 11 lines that had a mutated p53 gene were significantly more radiosensitive than those with wild-type p53 (Pekkola-Heino et al, 1996). Studies on other tumour types have yielded different results with some showing a significant association (Siles et al, 1996) and others not (Zaffaroni et al, 1995). In our work we have shown no relationship between SF2 and p53 expression, measured using immunohistochemistry in mixed cancers of the head and neck or in a subsite analysis of 31 oral cavity tumours ($r = 0.24$, $P = 0.20$). One of the principal functions of p53 is the induction of cell cycle arrest. As loss of wild-type expression is associated with cellular growth, a relationship might be expected between p53 expression and proliferation. This was not found either in our study or the work of others on head and neck cancer (Wilson et al, 1995). There are several possible reasons. First, it may be a methodological problem relating to the use of immunohistochemistry rather than mutational analysis. Immunohistochemistry using the DO7 antibody has been reported to have concordance with mutational analysis in 71% of head and neck cancers, while 14% were DO7-positive with no detected mutations and 15% had *TP53* mutations not detected by DO7 (Calzolari et al, 1997). Second, a lack of correlation between p53 expression and proliferation may relate to the variety of different effects mediated via p53. Third, it may be important to investigate a single tumour site within the head and neck. In support of the latter, Bourhis et al (1994) showed a significant correlation between p53 overexpression and short Tpot in 49 oropharyngeal cancers. Regarding the relationship between p53 and proliferation, more information would be gained by carrying out mutational analysis of *TP53* and by concentrating on a single tumour type. Although this is a limitation of the present study, our primary objective was to obtain radiosensitivity measurements on a large number of tumours, and correlate the radiosensitivity data with other biological parameters.

In summary, this work has highlighted the feasibility of carrying out multiple measurements of radiobiological parameters on patients with cancers of the head and neck. No correlations were seen between SF2 and measures of proliferation and p53 expression. All the patients included in this study were treated with radiotherapy, but follow-up times were too short to allow correlations with clinical outcome. However, in the future we will

*© 1999 Cancer Research Campaign*

examine the relationships between the various factors studied and radiation response in vivo.

### ACKNOWLEDGEMENTS

This work was supported by the King Gustav V Jubilee Clinical Cancer Research Foundation in Gothenburg and Berit and Carl Johan Wettergrens Foundation for Cancer Research in Sweden. Catharine West is supported by the Cancer Research Campaign (UK). The authors thank Ms Raghild Bernefors for her practical assistance. Discussion with Prof Jolyon Hendry is gratefully acknowledged.

### REFERENCES

Begg AC (1995) The clinical status of Tpot as a predictor? or why no tempest in the Tpot! *Int J Radiat Oncol Biol Phys* **32**: 1539–1541

Begg AC (1997) Individualization of radiotherapy. In: *Basic Clinical Radiobiology*, Steel GG (ed), pp 234–245. Arnold: London

Björk-Eriksson T, West CML, Karlsson E, Slevin NJ, James RD and Mercke C (1998) The in vitro radiosensitivity of human head and neck cancers. *Br J Cancer* **77**: 2371–2375

Bourhis J, Wilson G, Wibault P, Bosq J, Chavaudra N, Janot F, Luboinski B, Eschwege F and Malaise EP (1993) In vivo measurement of the potential doubling time by flow cytometry in oropharyngeal cancer treated by conventional radiotherapy. *Int J Radiat Oncol Biol Phys* **26**: 793–799

Bourhis J, Bosq J, Wilson GD, Bressac B, Talbot M, Leridant AM, Dendale R, Janin N, Armand JP and Luboinski B (1994) Correlation between p53 gene expression and tumour-cell proliferation in oropharyngeal cancer. *Int J Cancer* **15**: 458–462

Brachman DG, Beckett M, Graves D, Haraf D, Vokes E and Weichselbaum RR (1993) p53 mutation does not correlate with radiosensitivity in 24 head and neck cancer cell lines. *Cancer Res* **53**: 3667–3669

Bradford CR, Zhu S, Wolf GT, Poore J, Fisher SG, Beals T, McClatchey KD and Carey TE (1995) Overexpression of p53 predicts organ preservation using induction chemotherapy and radiation in patients with advanced laryngeal cancer. Department of Veterans Affairs Laryngeal Cancer Study Group. *Otolaryngol Head Neck Surg* **113**: 408–412

Bristow RG, Benchimol S and Hill RP (1996) The p53 gene as a modifier of intrinsic radiosensitivity: implications for radiotherapy. *Radiother Oncol* **40**: 197–223

Brock WA, Baker FL, Wike JL, Sivon SL and Peters LJ (1990) Cellular radiosensitivity of primary head and neck squamous cell carcinomas and local tumor control. *Int J Radiat Oncol Biol Phys* **18**: 1283–1286

Calzolari A, Chiarelli I, Bianchi S, Messerini L, Gallo O, Porfirio B and Mattiuz PL (1997) Immunohistochemical vs molecular biology methods. Complementary techniques for effective screening of p53 alterations in head and neck cancer. *Am J Clin Pathol* **107**: 7–11

Coucke PA, Pampalliona S, Paschoud N and Wilson GD (1998) Tpot versus labeling index: which way to go? In: *Progress in Radio-Oncology VI*, Kogelnik HD and Sedlmayer F (eds), pp. 531–538. Monduzzi Editore: Bologna, Italy

Eschwege F, Bourhis J, Girinski T, Lartigau E, Guichard M, Deblé D, Kepta L, Wilson GD and Luboinski B (1997) Predictive assays of radiation response in patients with head and neck squamous cell carcinoma: a review of the Institute Gustave Roussy experience. *Int J Radiat Oncol Biol Phys* **39**: 849–853

Field JK, Pavelic ZP, Spandidos DA, Stambrook PJ, Jones AS and Gluckman JL (1993) The role of the p53 tumor suppressor gene in squamous cell carcinoma of the head and neck. *Arch Otolaryngol Head Neck Surg* **119**: 1118–1122

Girinsky T, Bernheim A, Lubin R, Tavakoli-Razavi T, Baker F, Janot F, Wibault P, Cosset J-M, Duvillard P, Duverger A and Fertil B (1994) In vitro parameters and treatment outcome in head and neck cancers treated with surgery and/or radiation: cell characterization and correlations with local control and overall survival. *Int J Radiat Oncol Biol Phys* **30**: 789–794

Levine EL, Renehan A, Gossiel R, Davidson SE, Roberts SA, Chadwick C, Wilks DP, Potten CS, Hendry JH, Hunter RD and West CML (1995) Apoptosis, intrinsic radiosensitivity and prediction of radiotherapy response in cervical carcinoma. *Radiother Oncol* **37**: 1–9

Lydén E, Cvetkovska E, Westin T, Oldfors A, Soussi B, Gustavsson B and Edström S (1995) Effects of nandrolone propionate on experimental tumor growth and cancer cachexia. *Metabolism* **44**: 445–451

Narayana A, Vaughan ATM, Gunaratne S, Kathuria S, Walter SA and Reddy SP (1998) Is p53 an independent prognostic factor in patients with laryngeal carcinoma? *Cancer* **82**: 286–291

Nylander K, Stenling R, Gustavsson H, Zackrisson B and Roos G (1995) P53 expression and cell proliferation in squamous cell carcinomas of the head and neck. *Cancer* **75**: 87–93

Pekkola-Heino K, Joensuu H, Klemi P and Grenman R (1994) Relation of DNA ploidy and proliferation rate to radiation sensitivity in squamous carcinoma cell lines. *Arch Otolaryngol Head Neck Surg* **120**: 750–754

Pekkola-Heino K, Servomaa K, Kiuru A and Grenman R (1996) Increased radiosensitivity is associated with p53 mutations in cell lines derived from oral cavity carcinoma. *Acta Otolaryngol* **116**: 341–344

Raybaud-Diogene H, Fortin A, Morency R, Roy J, Monteil RA and Tetu B (1997) Markers of radioresistance in squamous cell carcinomas of the head and neck: a clinicopathological and immunohistochemical study. *J Clin Oncol* **15**: 1030–1038.

Sauter ER, Ridge JA, Gordon J and Eisenberg BL (1992) P53 overexpression correlates with increased survival in patients with squamous cell carcinoma of the tongue base. *Am J Surg* **164**: 651–653

Siles E, Villalobos M, Valenzuela MT, Nunez MI, Gordon A, McMillan TJ, Pedraza V and Ruiz de Almodovar JM (1996) Relationship between p53 status and radiosensitivity in human tumour cell lines. *Br J Cancer* **73**: 581–588

Spiessl B, Beahrs OH, Hermanet P, Hutter RVP, Scheibe O, Sobin LH and Wagner G (1990) *International Union against Cancer MNM Atlas Illustrated Guide to the TNM/pTNM Classification of Malignant Tumours*. Springer Verlag: Berlin

Tucker SL and Thames HD (1989) The effect of patient-to-patient variability on the accuracy of predictive assays of tumour response to radiotherapy: a theoretical evaluation. *Int J Radiat Oncol Biol Phys* **17**: 145–157

Warenius HM, Britten RA, Browning PG, Morton IE and Peacock JH (1994) Identification of human in vitro cell lines with greater intrinsic cellular radiosensitivity to 62.5 MeV (p→Be+) neutrons than 4 MeV photons. D. *Int J Radiat Oncol Biol Phys* **28**: 913–920

West CML (1995) Invited review: intrinsic radiosensitivity as a predictor of patient response to radiotherapy. *Br J Radiol* **68**: 827–837

West CML, Davidson SE, Roberts SA and Hunter RD (1997) The independence of intrinsic radiosensitivity as a prognostic factor for patient response to radiotherapy of carcinoma of the cervix. *Br J Cancer* **76**: 1184–1190

West CML, Cooper R, Davidson SE, Logue J, Hunter RD (1998) Optimising measurements of tumour radiosensitivity. In: *Progress in Radio-Oncology VI*, Kogelnik HD and Sedlmayer F (eds), pp. 517–524. Monduzzi Editore: Bologna, Italy.

Wilson GD, Richman PI, Dische S, Saunders MI, Robinson B, Daley FM and Ross DA (1995) p53 status of head and neck cancer: relation to biological characteristics and outcome of radiotherapy. *Br J Cancer* **71**: 1248–1252

Yin Y, Tainsky MA, Bischoff FZ, Strong LC and Wahl GM (1992) Wild-type p53 restores cell cycle control and inhibits gene amplification in cells with mutant p53 alleles. *Cell* **70**: 937–948

Zackrisson B, Gustafsson H, Stenling R, Flygare P and Wilson GD (1997) Predictive value of potential doubling time in head and neck cancer patients treated by conventional radiotherapy. *Int J Radiat Oncol Biol Phys* **38**: 677–683

Zaffaroni N, Benini E, Gornati D, Bearzatto A and Silvestrini R (1995) Lack of a correlation between p53 protein expression and radiation response in human primary cultures. *Stem Cells* **13**: 77–85

 *© 1999 Cancer Research Campaign*
